# Neuroinflammation, Oxidative Stress, Apoptosis, Microgliosis and Astrogliosis in the Cerebellum of Mice Chronically Exposed to Waterpipe Smoke

**DOI:** 10.3390/biomedicines11041104

**Published:** 2023-04-06

**Authors:** Naserddine Hamadi, Sumaya Beegam, Nur Elena Zaaba, Ozaz Elzaki, Mariam Abdulla Altamimi, Abderrahim Nemmar

**Affiliations:** 1 Department of Life and Environmental Sciences, College of Natural and Health Sciences, Zayed University, Abu Dhabi P.O. Box 144534, United Arab Emirates; 2 Department of Physiology, College of Medicine and Health Sciences, United Arab Emirates University, Al Ain P.O. Box 17666, United Arab Emirates; 3 Zayed Center for Health Sciences, United Arab Emirates University, Al Ain P.O. Box 17666, United Arab Emirates

**Keywords:** waterpipe smoking, cerebellum, neuroinflammation, oxidative stress, DNA damage

## Abstract

Waterpipe smoking (WPS) is prevalent in Asian and Middle Eastern countries and has recently gained worldwide popularity, especially among youth. WPS has potentially harmful chemicals and is associated with a wide range of adverse effects on different organs. However, little is known regarding the impact of WPS inhalation on the brain and especially on the cerebellum. Presently, we aimed at investigating inflammation, oxidative stress and apoptosis as well as microgliosis and astrogliosis in the cerebellum of BALB/C mice chronically (6 months) exposed to WPS compared with air-exposed mice (control). WPS inhalation augmented the concentrations of proinflammatory cytokines tumor necrosis factor, interleukin (IL)-6 and IL-1β in cerebellar homogenates. Likewise, WPS increased oxidative stress markers including 8-isoprostane, thiobarbituric acid reactive substances and superoxide dismutase. In addition, compared with the air-exposed group, WPS caused an increase in the oxidative DNA damage marker, 8-hydroxy-2′-deoxyguanosine, in cerebellar homogenates. Similarly, in comparison with the air group, WPS inhalation elevated the cerebellar homogenate levels of cytochrome C, cleaved caspase-3 and nuclear factor-κB (NF-κB). Immunofluorescence analysis of the cerebellum showed that WPS exposure significantly augmented the number of ionized calcium-binding adaptor molecule 1 and glial fibrillary acidic protein-positive microglia and astroglia, respectively. Taken together, our data show that chronic exposure to WPS is associated with cerebellar inflammation, oxidative stress, apoptosis, microgliosis and astrogliosis. These actions were associated with a mechanism involving NF-κB activation.

## 1. Introduction

Tobacco smoking is considered one of the major causes of preventable diseases and premature death worldwide [1]. According to a previous report, in the last two decades there was a dramatic shift in the methods of tobacco consumption [2]. While cigarette consumption has declined [3], the use of waterpipe smoking (WPS) has substantially increased [4]. The prevalence of WPS is growing particularly among young people, and it became an emerging global epidemic that requires action [5]. This increase in WPS use is attributed to the misconception that WPS is less toxic and less addictive than cigarette smoking (CS) as well as to the use of sweetened and flavored tobacco, which makes it more appealing to consumers [6]. 

Experimental and clinical studies, including our own, have reported that WPS induces multiple adverse health effects on various organs such as the heart, lungs and kidneys [7,8,9,10,11,12,13]. However, its effect on the brain is underexplored. 

It is well evidenced that CS is a risk factor in neurodegenerative diseases, and the associations between CS and neurological disorders such as stroke, Alzheimer’s disease, and multiple sclerosis have been established by various studies [14,15,16]. High levels of biomarkers for Alzheimer’s disease such as amyloid β42 levels and excessive oxidative stress and neuroinflammation have been reported in the brain of smokers [17]. Moreover, animal studies have revealed that CS exposure triggers multiple immune, inflammatory and oxidative responses in the brain that may play a crucial role in the pathogenesis of neurological diseases [18,19,20,21]. In the traumatic brain injury model, it was shown that there was CS-induced neuroinflammation and loss of blood–brain barrier integrity [22]. In addition, chronic exposure of rats to CS resulted in significant histological alterations, apoptosis, lipid peroxidation and mitochondrial dysfunctions in the brain [23]. Furthermore, the exposure of human premonocytic line U-937 and the rat insulinoma parental cell line RINm5F to tobacco smoke induced protein oxidation, DNA damage and cell death by apoptosis and necrosis [24]. 

In contrast, only a few experimental studies have explored the impact of WPS exposure on the brain. A clinical study has reported a reduced level of circulating brain-derived neutrophic factor in adolescent WPS smokers [25]. A prospective observational study has reported that exposure to WPS causes neuronal damage through the increase in cerebral blood flow rate, elevation in carboxyhemoglobin and S100 calcium-binding protein [26]. Furthermore, adverse effects on cognitive executive measures were recorded in humans following one session of WPS inhalation [27]. It has been reported that exposure to WPS induced inflammation in the mesocorticolimbic brain regions [28]. In addition, the occurrence of oxidative stress in the hippocampus and cognitive decline were shown in rats following WPS exposure [29,30]. 

The cerebellum represents about 10% of the mass of the brain [31]. It plays a crucial role in motor control, and recent studies have reported its involvement in cognition, emotions and social interaction [32,33,34,35]. A reduction in the cerebellar gray matter was shown in CS smokers compared to nonsmokers [36,37]. However, to the best of our knowledge, no study has investigated the effect of WPS exposure on the cerebellum. Therefore, in this study, we sought to examine the impact of chronic exposure to WPS in mice on cerebellar inflammation, oxidative stress, apoptosis, the expression of nuclear factor (NF)-κB and the occurrence of microgliosis and astrogliosis.

## 2. Materials and Methods

### 2.1. Animals and Treatments

This study was reviewed and approved by the United Arab Emirates University animal ethics committee, College of Medicine and Health Sciences, and experiments were performed according to protocols approved by the Institutional Animal Care and Research Advisory Committee. 

### 2.2. WPS Exposure

BALB/C mice (animal house facility, College of Medicine and Health Sciences, United Arab Emirates University) were housed in a conventional animal house and maintained on a 12 h light–dark cycle (lights on at 6:00 a.m.), humidity of 60% and controlled temperature (22 ± 1 °C). Animals had free access to water and food ad libitum. Animals were indiscriminately separated into air- and WPS-exposed groups after one week of familiarization to their conditions.

Mice were placed in soft restraints and connected to the exposure tower as described previously [38,39,40,41]. Using a nose-only exposure system connected to a waterpipe (InExpose System, Scireq, Montreal, QC, Canada), the animals were exposed to either WPS or air through their noses. Animals were exposed to mainstream WPS generated by commercially available apple-flavored tobacco (Al Fakher Tobacco Trading, Ajman, UAE). Tobacco was lit with instant light charcoal. Similar to its use in humans, the smoke from the waterpipe passed through the water first before it was drawn into the exposure tower which was controlled by a computerized system (In Expose System, Scireq, Montreal, QC, Canada). A computer-controlled puff was generated every minute, leading to a 2 s puff duration of WPS exposure followed by 58 s of fresh air. The duration of an exposure session was 30 min/day. Regarding the WPS group, mice were exposed to WPS 5 days/week for 6 months (mo), and control mice were exposed to air only [38,39,40,41]. 

### 2.3. Cerebellum Collection and Homogenates Preparation

At the end of the exposure period, WPS (*n* = 8) and air (*n* = 8) mice were sacrificed by decapitation. The brain was removed, and the cerebellum was immediately dissected out on ice, frozen in liquid nitrogen and stored at −80 °C until assayed. Cerebellar homogenates preparation for the measurement of markers of oxidative stress, inflammation and apoptosis were prepared as described before [42]. 

### 2.4. Measurement of the Concentrations of Tumor Necrosis Factor α (TNFα), Interleukin (IL)-6 and IL-1β

The concentrations of TNFα, IL-6 and IL-1β were measured using commercially available Elisa kits (Duo Set, R&D systems, Minneapolis, MN, USA). 

### 2.5. Quantification of Levels 8-Isoprostane, Thiobarbituric Acid Reactive Substances (TBARS) and Superoxide Dismutase (SOD)

The concentrations of 8-isoprostane were assayed according to the protocols described by the manufacturer (Cayman Chemicals, Ann Arbor, MI, USA). NADPH-dependent membrane lipid peroxidation was measured as TBARS, using malondialdehyde as the standard (Sigma-Aldrich Fine Chemicals, St. Louis, MO, USA). The activity of the antioxidant enzyme SOD was quantified as per the vendor’s protocols (Cayman Chemicals, Ann Arbor, MI, USA). 

### 2.6. Assessment of 8-Hydroxy-2′-Deoxyguanosine (8-OHdG), Cytochrome C, Cleaved Caspase-3, Nuclear Factor-κB (NF-κB) and Phosphorylated (Phospho)-NF-κB 

Using commercially available ELISA kits, we measured in cerebellum homogenates the levels of 8-OHdG (Cusabio Biotech Co., Ltd., Wuhan, China), cytochrome C (R&D Systems, Minneapolis, MN, USA), cleaved caspase-3 (R&D Systems, Minneapolis, MN, USA), NF-κB and phospho-NF-κB (Cell Signaling Technology, Danvers, MA, USA) as described previously [43,44,45].

### 2.7. Immunofluorescence Labeling

For the immunofluorescence analysis we used a separate set of animals; at the end of 6 mo exposure to WPS, mice of each group WPS (*n* = 5) and air (*n* = 5) were deeply anesthetized with sodium pentobarbital (35 mg/kg, i.p) and perfused with 20 mL of phosphate-buffered saline followed by 100 mL of freshly prepared 4% formaldehyde as a fixative. The cerebellum was post-fixed with formalin and embedded with paraffin. Using microtome (Leica, RM2125RT, Wetzlar, Germany), 1 μm thick sagittal sections of the cerebellum were cut and mounted on gelatin-coated slides. Sections were deparaffinized with xylene and rehydrated with a graded series of ethanol, and heat-induced antigen retrieval was performed in citrate buffer (pH 6.0) using the microwave.

Double immunofluorescence labeling was achieved by incubation at 4 °C overnight with the primary antibodies. We used rabbit polyclonal anti-Iba-1 (cat#019-19741, Wako, Osaka, Japan, 1:1500) for microglia, guinea pig monoclonal anti-neuronal nuclear antigen antibody (Neun) (cat#ABN90P, MerckMillipore, Darmstadt, Germany, 1:1500) for neurons and rabbit polyclonal anti-GFAP (cat#Z0334, Dako, Glostrup, Denmark, 1:500) for astroglia. The next day, sections were washed three times in PBS, followed by incubation for 1 h at room temperature with secondary antibodies: donkey anti-rabbit conjugated to Alexa 488 (cat#A32790, Invitrogen, Rockford, USA, 1:200) and donkey anti-guinea pig conjugated to Rhodamine (cat#706-295-148, Jakson, PA, USA, 1:100). Next, sections were incubated with 4,6-diamidino-2-phenylindole (DAPI) for nuclei counterstaining for 5 min and then were washed in PBS three times. Slides were then cover-slipped with Fluoroshield mounting medium (cat#ab104135, Abcam, Waltham, MA, USA, 1:100). Images were acquired using an EVOS M5000 microscope.

For quantification of Iba-1,GFAP and NEUN/DAPI positive microglia, astroglia and neurons, respectively, three sections of the cerebellum from each animal were used; five animals from each group. Cell counting was performed at 20X magnification in the whole sections of the cerebellum with ImageJ software (NIH, Bethesda, MY, USA).

### 2.8. Statistical Analysis

The statistical analysis was performed using GraphPad Prism (version 7; GraphPad Software Inc., San Diego, CA, USA). Data were tested using the unpaired *t*-test for differences between the two groups. The results are expressed as the mean ± SEM, and *p*-values of <0.05 were considered to be significantly different.

## 3. Results

### 3.1. Effect of Chronic Exposure to WPS on the Concentrations of TNFα, IL-6 and IL-1β in the Cerebellum

**Figure 1:**
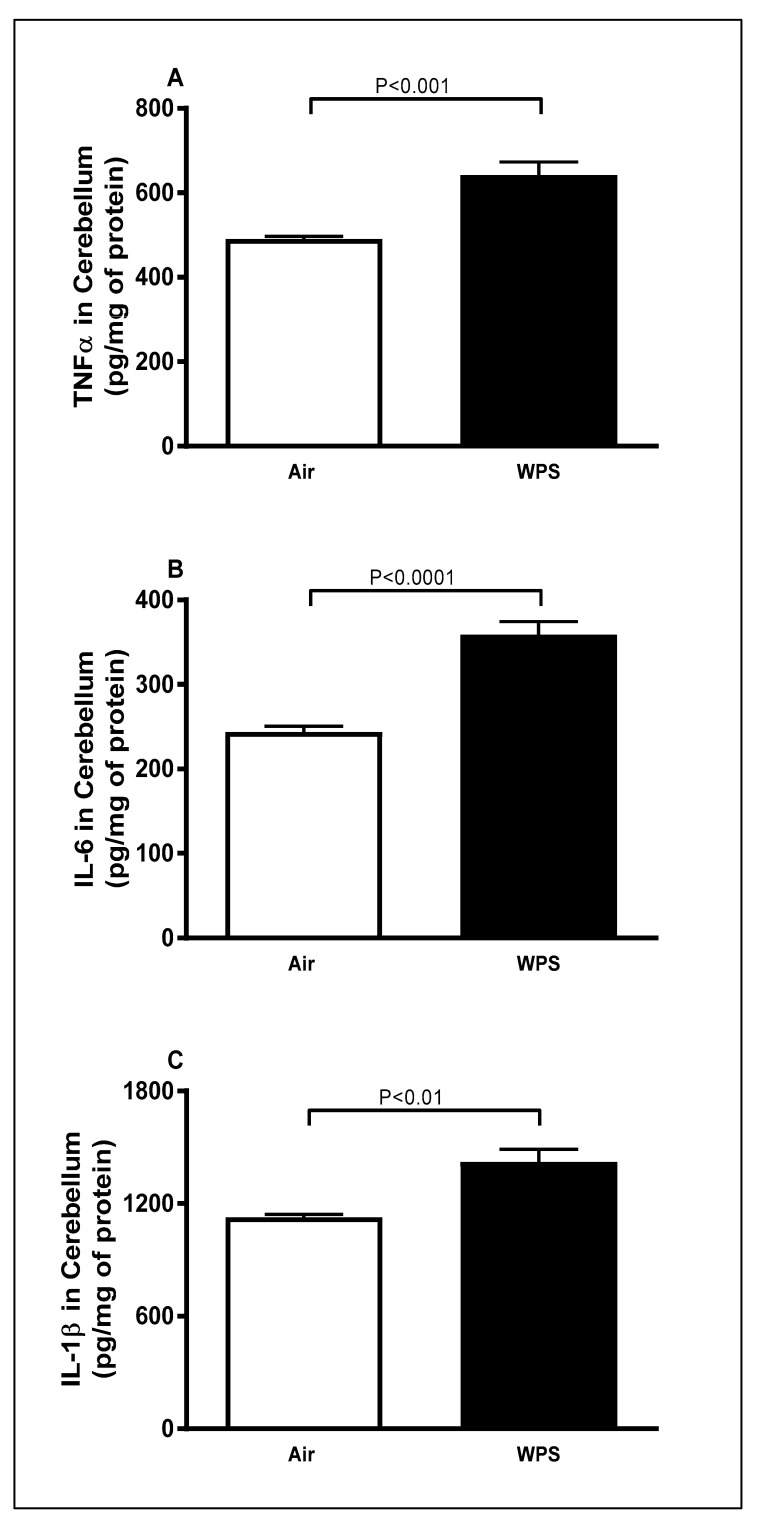
Tumor necrosis factor α (**A**), interleukin-6 (**B**) and interleukin-1β (**C**) concentrations in the cerebellar homogenates of mice exposed to either air or waterpipe smoke (WPS) for 6 months. Data are mean ± SEM (*n* = 7–8).

Figure 1 shows that, compared to the air-exposed group, WPS exposure for 6 mo caused a significant increase in the concentrations of TNFα (Figure 1A, *p* < 0.001), IL-6 (Figure 1B, *p* < 0.0001) and IL-1β (Figure 1C, *p* < 0.01) in the cerebellar homogenates. 

### 3.2. Effect of Chronic Exposure to WPS on the Concentrations of 8-Isoprostane and TBARS and Activity of SOD in the Cerebellum

**Figure 2:**
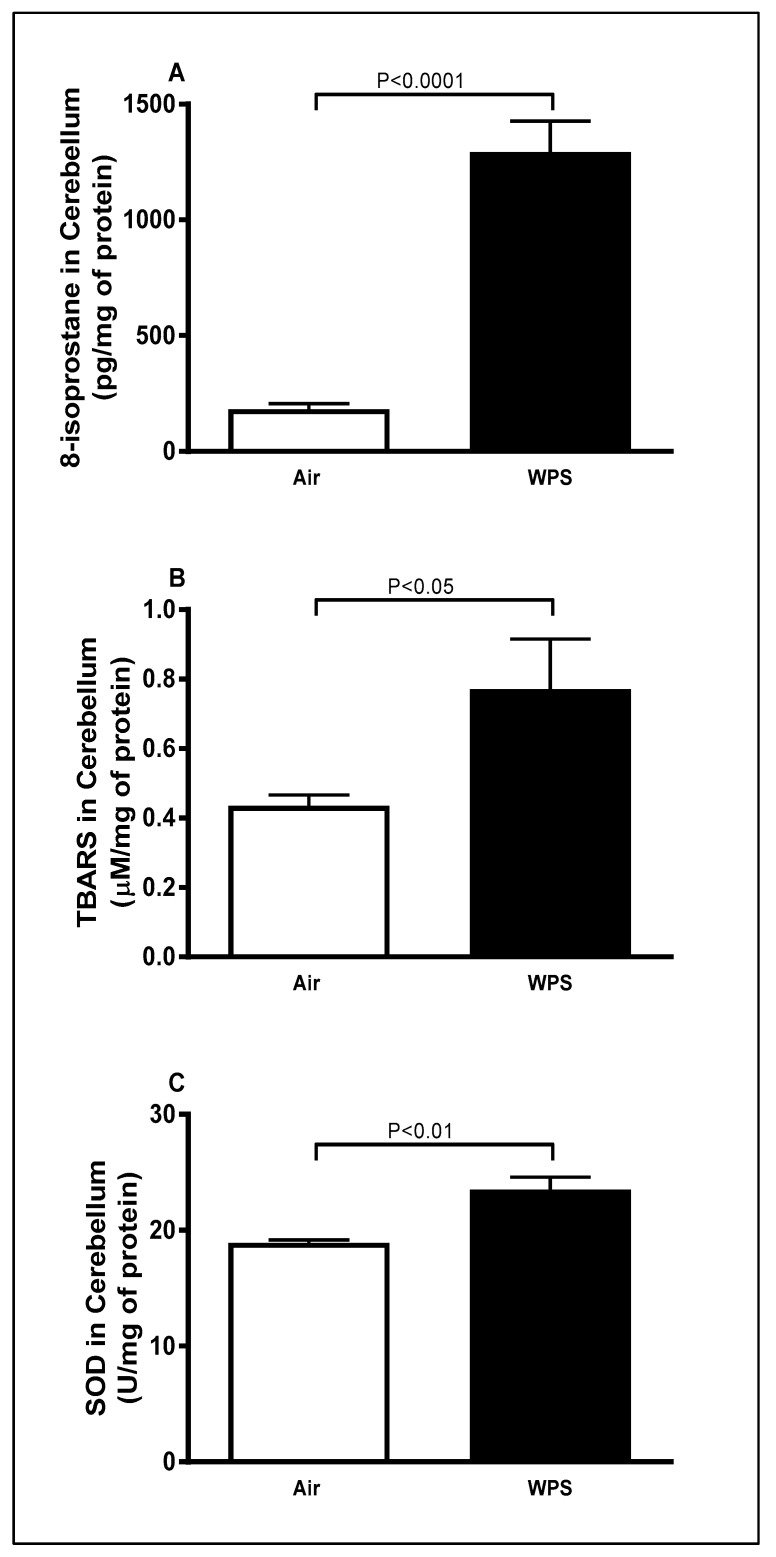
8-isoprostane (**A**), thiobarbituric acid reactive substances (**B**) and superoxide dismutase (**C**) levels in the cerebellar homogenates of mice exposed to either air or waterpipe smoke (WPS) for 6 months. Data are mean ± SEM (*n* = 7–8).

Compared to the air-exposed group, WPS inhalation for 6 mo induced a significant increase in the concentrations of 8-isoprostane (Figure 2A, *p* < 0.0001), TBARS (Figure 2B, *p* < 0.05) and activity of SOD (Figure 2C, *p* < 0.01) in the cerebellar homogenates. 

### 3.3. Effect of Chronic Exposure to WPS on the Concentrations of 8-OHdG in the Cerebellum

**Figure 3:**
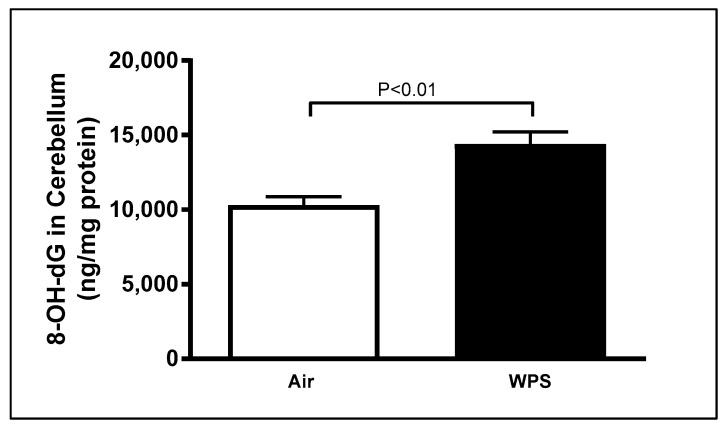
8-hydroxy-2′-deoxyguanosine (8-OH-dG) concentrations in the cerebellar homogenates of mice exposed to either air or waterpipe smoke (WPS) for 6 months. Data are mean ± SEM (*n* = 7–8).

As illustrated in Figure 3, there was a significant (*p* < 0.01) increase in the concentrations of 8-OHdG, a marker of oxidative DNA damage, in the cerebellar homogenates following WPS exposure for 6 mo compared with the air-exposed group (Figure 3).

### 3.4. Effect of Chronic Exposure to WPS on the Levels of Cleaved Caspase-3 and Cytochrome C in the Cerebellum

**Figure 4:**
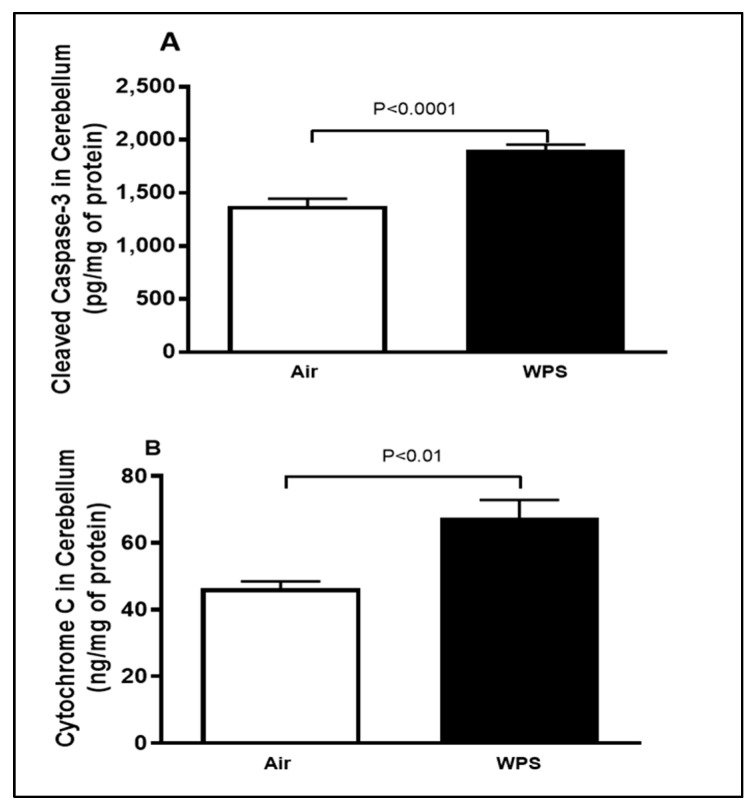
Cleaved caspase-3 (**A**) and cytochrome C (**B**) concentrations in the cerebellar homogenates of mice exposed to either air or waterpipe smoke (WPS) for 6 months. Data are mean ± SEM (*n* = 7–8).

Figure 4 illustrates that WPS exposure for 6 mo induced a significant increase in the levels of cleaved caspase-3 (*p* < 0.0001) and cytochrome C (*p* < 0.01) in the cerebellar homogenates compared with the air-exposed group.

### 3.5. Effect of Chronic Exposure to WPS on the Expression of NF-κB and Phospho-NF-κB in the Cerebellum

**Figure 5:**
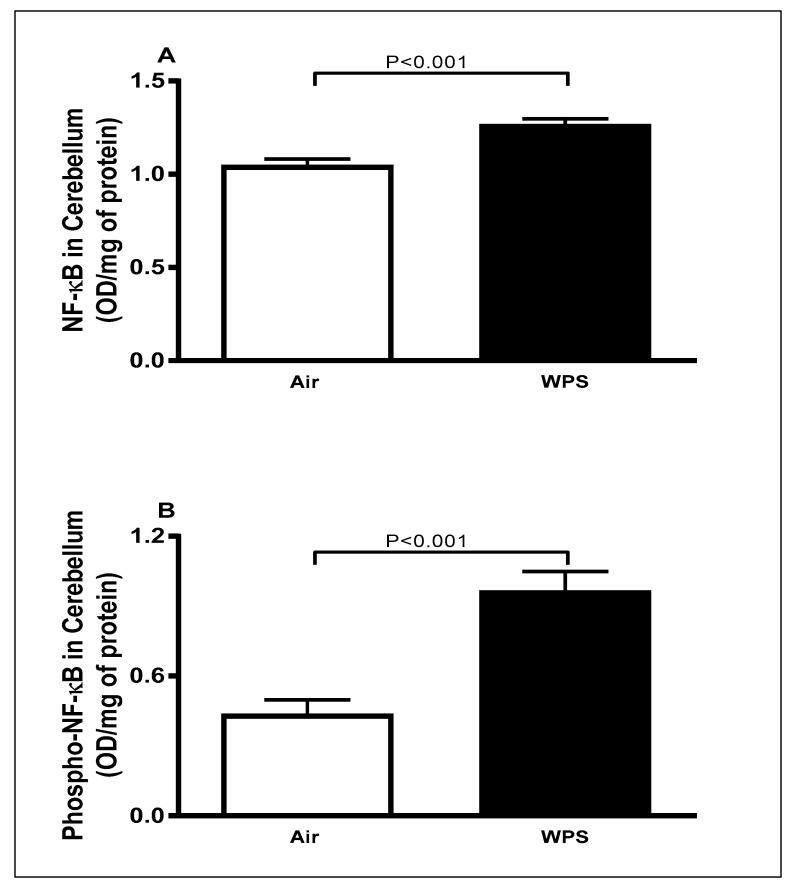
The total concentrations of nuclear factor kappa B (NF-κB) (**A**) and phosphorylated-NF-κB (**B**) in the cerebellar homogenates of mice exposed to either air or waterpipe smoke (WPS) for 6 months. Data are mean ± SEM (*n* = 7–8).

The total concentrations of NF-κB and phospho-NF-κB in the cerebellar homogenates are shown in Figure 5. WPS exposure for 6 mo resulted in a significant elevation (*p* < 0.001) in the total concentrations of NF-κB (Figure 5A) and phospho-NF-κB (Figure 5B) compared with the air-exposed group. 

### 3.6. Effect of Chronic Exposure to WPS on Iba-1 Labeling and Quantification of Iba-1-Positive Microglia in the Cerebellum

**Figure 6:**
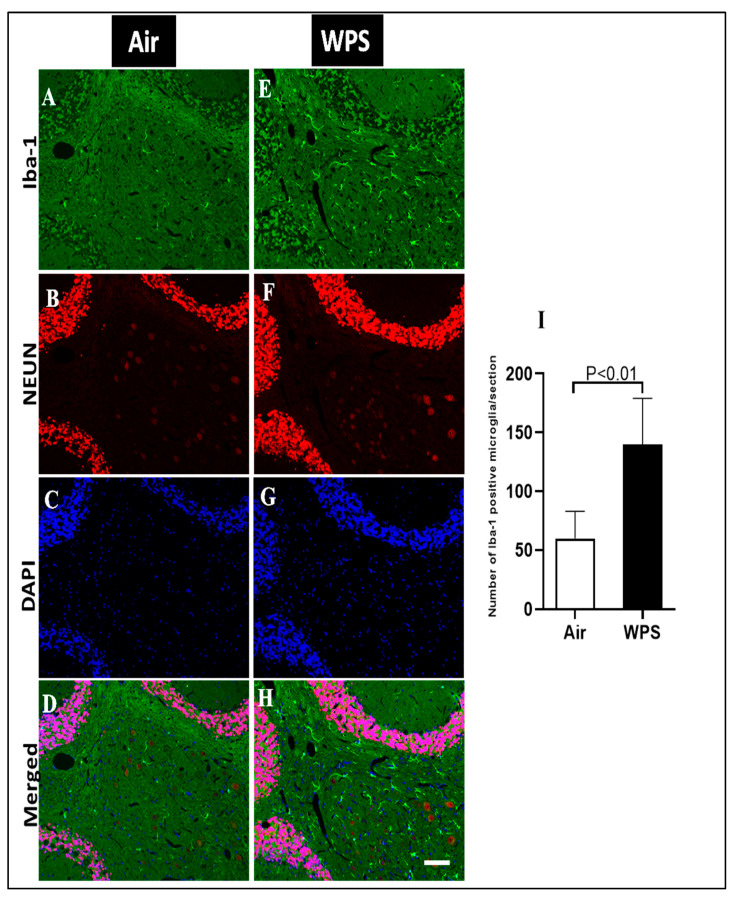
Representative images of double-immunofluorescence staining for anti-ionized calcium-binding adaptor molecule 1 (Iba-1, green), anti-neuronal nuclear antigen antibody (red) and 4, 6-diamidino-2-phenylindole (blue as a nuclei counterstain) of sagittal sections of the cerebellum of mice exposed to either air or waterpipe smoke (WPS) for 6 months. (**A**–**D**) Representative images of air-exposed mice showing ramified, resting microglia in the white matter of the cerebellum. (**E**–**H**) Representative images of WPS-exposed mice showing increased Iba-1 immunoreactivity in the white matter of the cerebellum. Scale bars = 50 µm, (*n* = 5). (**I**) Bar graph showing cell counting of Iba-1 positive microglia that revealed a significant increase in the number of microglial cells in the cerebellum of mice exposed either to air or WPS for 6 months. Data are mean ± SEM (*n* = 5).

The double immunofluorescence staining of microglia (Iba-1, green), neurons (NEUN, red) and the counterstain for nuclei (DAPI, blue) of sagittal sections of the cerebellum after 6 mo WPS exposure revealed an increase in the immunoreactivity of Iba-1 in different areas, indicating the occurrence of microgliosis (Figure 6E,H). In contrast, representative images of air-exposed mice showed a lower immunoreactivity of Iba-1 where the microglia appeared in their resting state with small cell bodies and thin processes in the white matter of the cerebellum (Figure 6A,D). The quantitative analysis showed that the number of Iba-1-positive microglia in the cerebellum of WPS-exposed mice was significantly higher (*p* < 0.01) compared with the air-exposed group (Figure 6I). 

**Figure 7:**
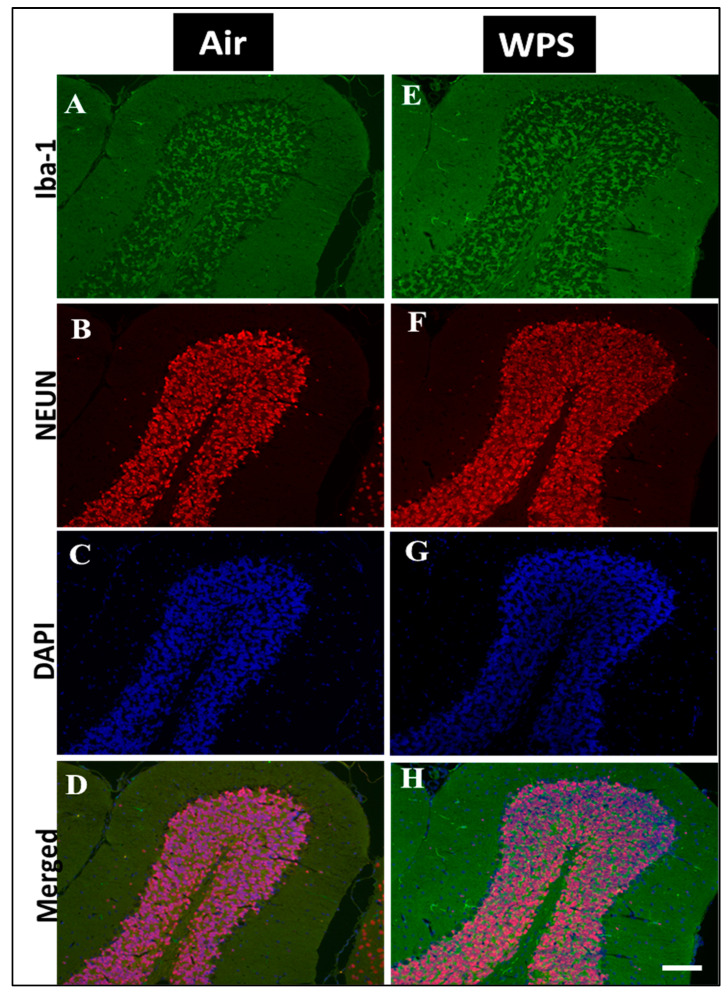
Representative images of double-immunofluorescence staining for Iba-1 (Iba-1, green), NEUN (red) and DAPI (blue as a nuclei counterstain) of sagittal sections of the cerebellum of mice exposed to either air or waterpipe smoke (WPS) for 6 months. (**A**–**D**) Representative images of air-exposed mice showing ramified, resting microglia in the granular layer of the cerebellum. (**E**–**H**) Representative images of WPS-exposed mice showing increased Iba-1 immunoreactivity in the granular layer of the cerebellum. Scale bars = 50 µm, (*n* = 5).

The examination of the Iba-1 labeling of the cerebellum showed a slight increase in the number of Iba-1 positive microglia in the granular layer (Figure 7E,H) of the cerebellum after WPS exposure compared with the air-exposed group (Figure 7A,D). 

### 3.7. Effect of Chronic Exposure to WPS on the GFAP Labeling and Quantification of GFAP-Positive Astroglia in the Cerebellum

**Figure 8:**
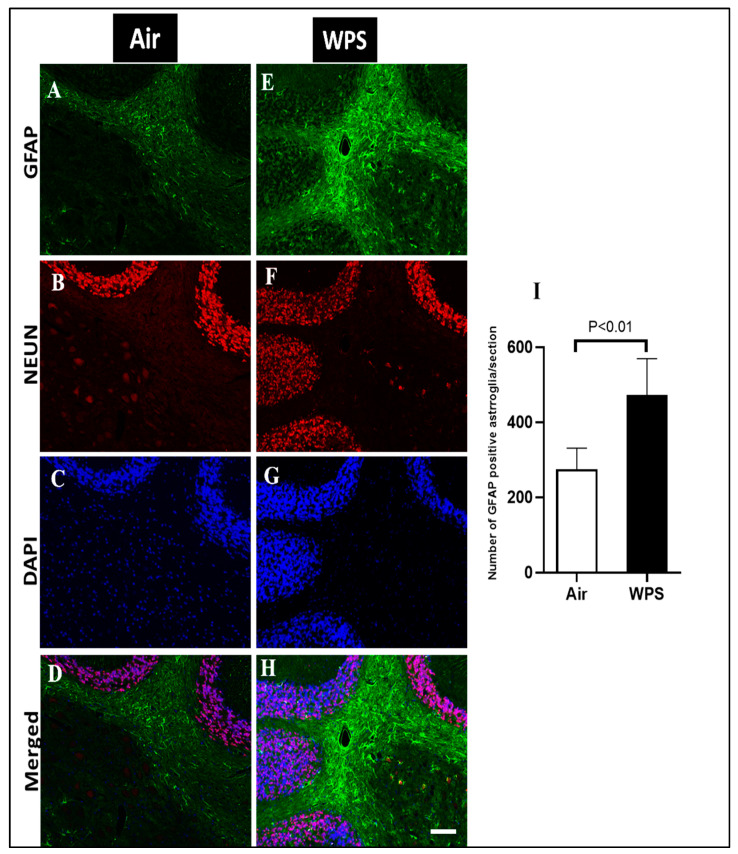
Representative images of double-immunofluorescence staining for GFAP (green), NEUN (red) and DAPI (blue as a nuclei counterstain) of sagittal sections of the cerebellum of mice exposed to either air or waterpipe smoke (WPS) for 6 months. (**A**–**D**) Representative images of air-exposed mice showing astrocytes with small cell bodies and thin processes in the white matter of the cerebellum. (**E**–**H**) Representative images of WPS-exposed mice showing increased GFAP immunoreactivity in the white matter of the cerebellum. Scale bars = 50 µm, (*n* = 5). (**I**) Bar graph showing the cell counting of GFAP positive astroglia that revealed a significant increase in the number of astroglia cells in the cerebellum of mice exposed either to air or waterpipe smoking for 6 months. Data are mean ± SEM (*n* = 5).

**Figure 9:**
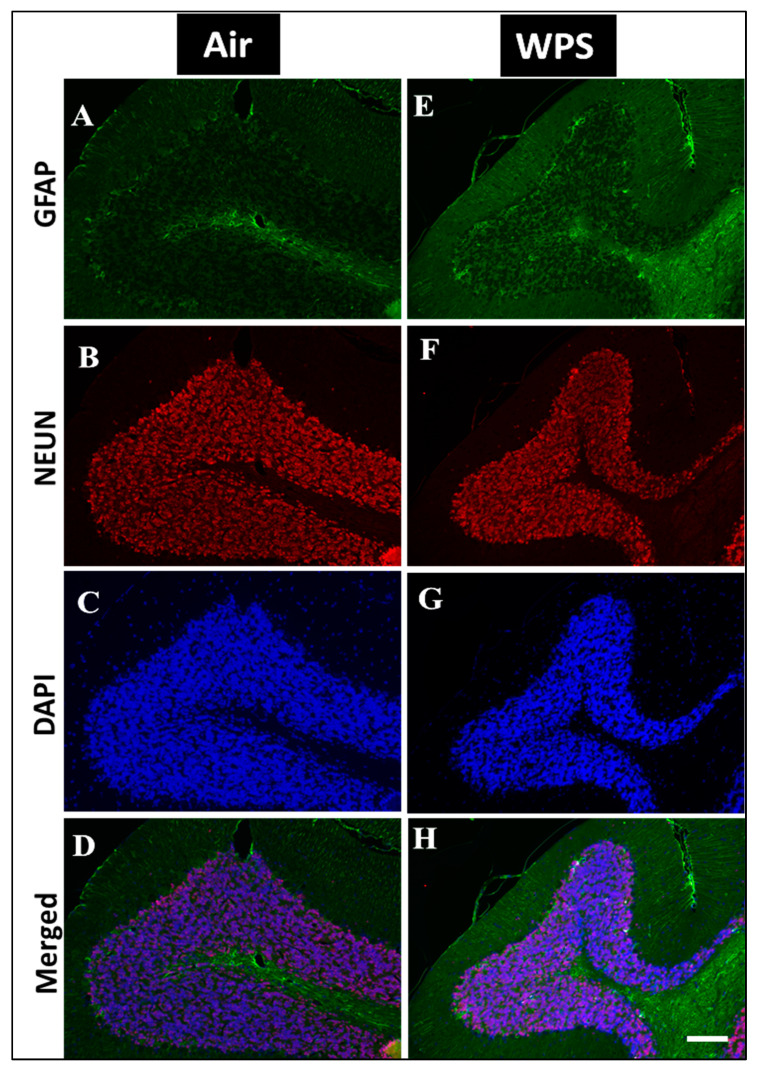
Representative images of double-immunofluorescence staining for GFAP (green), NEUN (red) and DAPI (blue as a nuclei counterstain) of sagittal sections of the cerebellum of mice exposed to either air or waterpipe smoke (WPS) for 6 months. (**A**–**D**) Representative images of air-exposed mice showing astrocytes with small cell bodies and thin processes in the granular layer of the cerebellum. (**E**–**H**) Representative images of WPS-exposed mice showing increased GFAP immunoreactivity in the granular layer of the cerebellum. Scale bars = 50 µm. Data are mean ± SEM (*n* = 5).

In order to examine the effect of WPS exposure for 6 mo on GFAP immunoreactivity in the cerebellum, a double immunofluorescent labeling of astroglia (GFAP, green), neurons (NEUN, red) and the counterstain for nuclei (DAPI, blue) was performed. Activated astrocytes underwent morphological changes and appeared hypertrophic and showed intense GFAP immunoreactivity in the white matter of the cerebellum of WPS-exposed mice (Figure 8E,H). In the air-exposed group, the astrocytes appeared with thin cell bodies and short processes (Figure 8A,D). Figure 8I shows that the exposure to WPS for 6 mo resulted in a significant increase (*p* < 0.01) in the number of GFAP-positive astrocytes in the cerebellum compared with the air-exposed group. Moreover, Figure 9E,H shows more GFAP immunoreactivity in the granular layer after WPS exposure in comparison with the air-exposed group (Figure 9A,D). 

### 3.8. Effect of Chronic Exposure to WPS on the NEUN/DAPI Labeling and Quantification of Neurons in the Cerebellum

**Figure 10:**
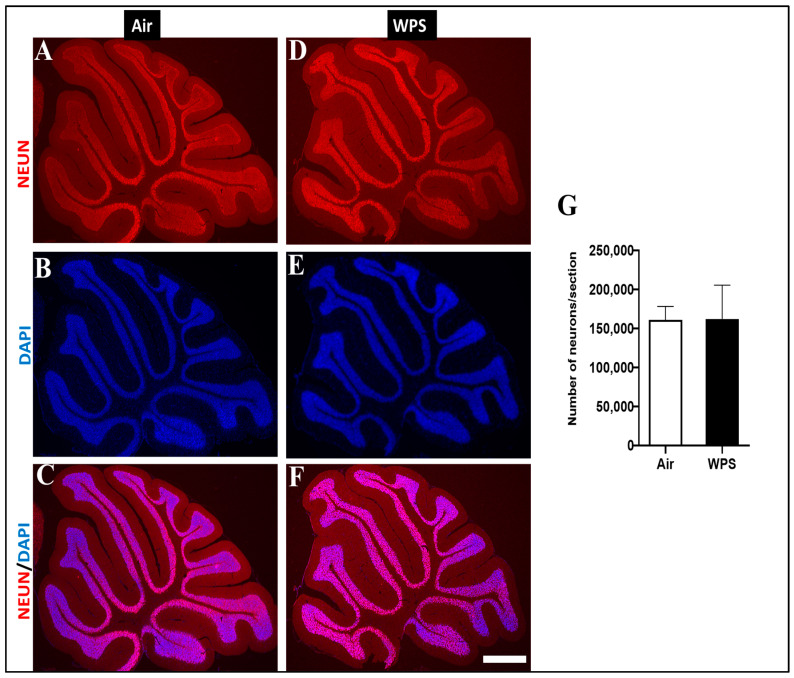
Representative images of double-immunofluorescence staining for anti-neuronal nuclear antigen antibody (red) and 4, 6-diamidino-2-phenylindole (blue as a nuclei counterstain) of sagittal sections of the cerebellum of mice exposed to either air or waterpipe smoke (WPS) for 6 months. (**A**–**C**) Representative images of the cerebellum of air-exposed mice. (**D**–**F**) Representative images of the cerebellum of WPS-exposed mice. Scale bars = 100 µm. (**G**) Bar graph showing neuron counting, demonstrating no statistical difference in the number of neurons in the cerebellum of mice exposed either to air or WPS for 6 months. Data are mean ± SEM (*n* = 5).

The analysis of NEUN/DAPI labeling of the cerebellum revealed no statistical difference in the number of neurons of mice exposed to WPS compared with the control group (Figure 10).

## 4. Discussion

Waterpipe smoking is becoming a popular trend worldwide. Research in this area has mostly highlighted the cardiovascular and respiratory pathophysiologic effects of WPS. However, little is known regarding the role of WPS in causing biochemical and histopathological alterations in the brain and, more specifically, in the cerebellum. This study is the first of its kind that focused on the effects of WPS on the cerebellum in rodents. The data of the current study show that long-term exposure to WPS is associated with inflammation, oxidative stress, DNA damage, apoptosis, microgliosis and astrogliosis in the cerebellum.

Several studies have reported a consistent association between smoking and increased risk of dementia, including Alzheimer’s disease (AD), vascular dementia and cognitive decline [46,47,48]. In addition, one of the hallmarks of neurodegenerative diseases is the increase in the concentration of proinflammatory markers in the brain [49]. The present study supports previous observations on the fact that neuroinflammatory responses are associated with smoking. Our data show that WPS exposure for 6 mo induced neuroinflammation, as indicated by the significant augmentation in the concentrations of proinflammatory cytokines TNFα, IL-6 and IL-1β in the cerebellar homogenate. In agreement with our results, WPS inhalation in rats was found to cause a significant increase in the expression of TNFα mRNA in mesocorticolimbic brain regions [28]. Moreover, high expression of proinflammatory cytokines in the brain was seen following 3–6 weeks of CS exposure in rats and mice [19,22]. It has been shown that the treatment with one of the most potent ingredients present in CS [48] and WPS [50], such as Benzo[a]pyrene diol epoxide (BPDE), led to an augmentation in mRNA and protein levels of the highly inducible inflammation factor cyclooxygenase-2 in the cortical cells of rats [51]. In support of the latter, treatment with 4-N-methyl-N-nitrosamino-1-(3-pyridyl)-1-butanone (NNK), a major nitrosamine formed in tobacco smoke, has been shown to induce a significant increase in the expression of TNFα, IL-6 and IL-12 both in vivo and in vitro [52].

Oxidative stress is a major trigger of cell damage, and it is well-established that it plays a role in the pathogenesis of various neurological disorders [53,54,55]. Our data show that long-term exposure to WPS caused a substantial increase in the concentrations of markers of lipid peroxidation TBARS and 8-isporstane. The increase of TBARS and 8-isporstane is an indication of the development of oxidative stress and the occurrence of lipid peroxidation in the cerebellum. Moreover, our results showed an increase in the activity of SOD. This increase might be considered to be a compensatory mechanism aiming at counterbalancing the ongoing oxidative injury induced by nose-only WPS inhalation in the cerebellum. Using whole body exposure system to WPS, Alzoubi et al. [29] reported the occurrence of oxidative stress in the hippocampus evidenced by a decrease in the activities of key antioxidant enzymes including glutathione peroxidase, catalase and SOD indicating a consumption of these antioxidants in the course of combatting WPS-induced oxidative damage. Inflammation and oxidative stress in cells often target the DNA. It is well known that repeated oxidative injury of the DNA is implicated in the pathogenesis of numerous diseases such as chronic pulmonary diseases, atherosclerosis and neurodegenerative disorders [56,57,58]. Our study showed that WPS-exposed mice had higher levels of the oxidative DNA damage marker 8-OHdG. The latter is the predominant form of free radical-induced oxidative lesions in nuclear and mitochondrial DNA [59]. This observation is in agreement with a previous study that showed high levels of 8-OHdG in the brain of rats exposed chronically to passive smoking [47].

Inflammation and oxidative stress in the brain are the main causes of DNA damage and cell death leading to neurodegeneration, which is the predominant phenomenon that underlies the symptoms of multiple human neurological disorders [60]. Cell death frequently implies the initiation of apoptosis via caspase activation [60]. Hence, caspase-3 is commonly used as a marker of apoptosis. In this work, we found that WPS-exposed mice displayed a significant augmentation in cleaved caspase-3. This observation is consistent with a study that showed that exposure to CS (6 cigarettes/twice a day) for 57 days significantly increased the immunoreactivity of cleaved caspase-3 in the white matter but not in the granular layer of the cerebellum of rats [61].

Cytochrome C is involved in both cellular energy and apoptosis [62]. Our data showed that 6 mo exposure to WPS induced a significant increase in the activity of cytochrome C. In line with our observation, Hosseini et al. [63] showed that the incubation of rat mitochondria with different concentrations of CS extract triggered the collapse of mitochondrial membrane potential, mitochondrial swelling and outer membrane rupture that resulted in the release of cytochrome C. We have previously shown that WPS inhalation in mice caused an elevation of cytochrome C in the kidney and testicular tissues [44,64]. 

In an attempt to investigate the underlying mechanism of the increase in oxidative stress and neuroinflammation observed in the present work, we measured the transcription factor NF-κB. The latter is commonly known as a critical regulator of both oxidative stress and inflammatory responses through the regulation of oxidative stress and numerous proinflammatory genes such as cytokines [65,66,67]. Our data show that WPS induced a significant increase in the levels of NF-κB. Consistent with our results, it has recently been demonstrated that WPS exposure for 4 weeks led to a significant increase in the expression of mRNA levels of NF-κB in different areas of the brain of rats including the prefrontal cortex, nucleus accumbens and ventral tegmental area [28]. We have previously reported that exposure to WPS in mice induced a marked increase in the expression of NF-κB in various organs including the heart, lungs and testicles [11,64,68]. Additional studies involving an inhibitory approach through repressing the NF-κB gene and/or the use of NF-κB knockout mice are needed to establish whether NF-κB plays a critical role in the observed effects. 

Microglia are the smallest cells in the brain, and the resident macrophages in the brain [69], and continuously monitor the extracellular environment of neurons [70]. In response to neuronal insults, microglia cells change their morphology and their numbers increase noticeably, which is termed microgliosis [71]. Our study revealed a marked increase in the number of microglia in the granular layer and more abundantly in the white matter. It has been demonstrated that CS condensate accelerates the activation of microglia in the experimental autoimmune model [72]. In support of the latter, immunohistochemical staining revealed a massive microglial and astrocyte activation in the brain of mice following exposure to the tobacco carcinogen NNK [52].

Astrocytes are the most abundant cell type within the central nervous system [73]. They play a critical role in the health of the central nervous system by maintaining structural support, formation of the blood-brain barrier, neuronal metabolism and neurotransmitter synthesis [74]. In order to investigate the activation of astrocytes in response to various injuries, GFAP has been used as a primary marker for astrocytes [75]. Our results revealed an activation of the astrocytes all over the cerebellum and, more intensely, at the level of the white matter where we observed a hypertrophy of astrocytes. This observation is consistent with a prior study performed on rats demonstrating that gestational maternal exposure of rats to nicotine induced an elevation in the expression of GFAP in the cerebellum of the offspring at puberty [76]. Fuller et al. [77] reported an increase in the GFAP immunoreactivity in the cerebellum in adult rats exposed to CS for 3 weeks. 

It has been shown that activated microglia promote stimulation of astrocytes through the secretion of IL-1β, which may work to increase the production of other cytokines such as IL-6, mainly from astrocytes [78]. In addition, it has been suggested that upon activation, astrocytes might play a potential role in the exacerbation of the neuronal and structural damage through the release of cytokines, chemokines, nitric oxide and reactive oxygen species, all of which can induce and potentiate inflammation [79]. The latter can cause DNA damage and apoptosis [80,81]. Therefore, we can speculate that the cerebellar inflammation and oxidative stress seen presently could plausibly be linked, at least partly, to the observed microgliosis and astrogliosis. Further experimental work is required to clarify this point.

In the current work, we did not correlate the observed biochemical and histological findings with cerebellar motor functions. Therefore, further studies are required to address this point using behavioral tests such as the rotarod test (to evaluate motor function and motor coordination) and treadmill gait analysis (to measure motor performance in mice by assessing quantitatively the gait). 

In summary, we can conclude that our findings demonstrate for the first time, that chronic exposure to WPS triggers inflammatory responses, oxidative stress, DNA damage and apoptosis through mechanisms associated with NF-κB activation. These biochemical changes were accompanied with a marked astrogliosis and microgliosis in the cerebellum. Therefore, our data provide supporting evidence that WPS is a major risk factor for neuropathological alterations. 

## Data Availability

The data that support the findings of this study are available from the corresponding author, Abderrahim Nemmar, upon reasonable request.
